# Persistent Vulvar Itch Unresponsive to Treatment: A Case of Vulvar Schistosomiasis Caused by *Schistosoma mansoni* and a Brief Review of Literature

**DOI:** 10.1155/2023/9913905

**Published:** 2023-10-18

**Authors:** Anthony Gyening-Yeboah, Solomon E. Quayson

**Affiliations:** ^1^Department of Pathology, Korle Bu Teaching Hospital, Accra, Ghana; ^2^University of Ghana Medical School, Accra, Ghana

## Abstract

**Background:**

Vulvar schistosomiasis is a female genital schistosomiasis (FGS), which occurs because of the damage caused by the presence of schistosome ova within the vulva. FGS is mostly misdiagnosed as a sexually transmitted infection. There is no reported case of vulvar schistosomiasis from *Schistosoma mansoni* in an immunocompetent or immunocompromised person in Ghanaian medical literature; however, there is a reported case of *S. haematobium* in an immunocompromised person. This is the first case of vulvar schistosomiasis from *S. mansoni* infection in an immunocompromised person. This case report discusses the need to consider vulvar schistosomiasis in patients with itchiness of the vulva. *Case Presentation*. A sixty-nine-year-old married woman presents with a persistent vulvar itch that is unresponsive to treatment. A clinical diagnosis of vulvar lichen planus unresponsive to medical therapy was made. A histopathological diagnosis of vulvar schistosomiasis was, however, made. Ziehl–Neelsen stain revealed the ova of *Schistosoma mansoni*. Symptoms resolved on administration of oral praziquantel.

**Conclusion:**

Vulvar schistosomiasis must be considered in clinical history-taking and investigation of signs and symptoms related to itchiness of the vulva. Ziehl–Neelsen staining is a helpful histopathology armamentarium to determine the species of schistosome ova.

## 1. Introduction

Schistosomiasis or bilharzia is an acute and chronic parasitic infection caused by Schistosoma [1, 2]. In acute infections, schistosomiasis presents as swimmer's itch, and as Katayama syndrome, which was first described in 1847 in Japan [1, 3–5]. Chronic schistosomiasis presents as granulomatous inflammation against schistosome ova [1, 4, 5]. *Schistosoma haematobium*, *S. mansoni*, *S. japonicum*, *S. intercalatum*, and *S. mekongi* are 5 schistosome species that are known to cause significant human infections [1, 2]; however, *S. haematobium* and *S. mansoni* are mainly implicated in the disease burden in sub-Saharan Africa [1, 6]. Schistosomiasis has been with humanity from ancient civilizations till the present day [3]. The presence of schistosomiasis amongst human beings is not unusual because of the consistent use of water [7–10].

Vulvar schistosomiasis (VS) is usually caused by *S. haematobium* and occasionally by *S. mansoni* [11]. It is not uncommon to find vulvar schistosomiasis in communities with high *S. haematobium* and *S. mansoni* endemicity [12]; however, to the best of our knowledge, there is no reported case of vulvar schistosomiasis caused by *S. mansoni* in Ghanaian medical literature and no reported case of vulvar schistosomiasis in an immunocompetent person. There is, however, a recently reported case of vulvar schistosomiasis in an immunocompromised person with human immunodeficiency virus (HIV) infection in Ghana [13]. A retrospective study by Der et al. showed that there was no reported case of vulvar schistosomiasis from 2004 to 2011 at the KBTH, Accra, the biggest health facility in Ghana [14]. Also, only 2 (1.3%) cases out of the 151 cases of tissue schistosomiasis reviewed were caused by *S. mansoni* [14]. The treatment of choice for vulvar schistosomiasis is 40 mg/kg of praziquantel [1, 15, 16]. Primary and secondary preventative measures are preferred because schistosomiasis-related complications cannot be adequately resolved with praziquantel [16]. We present a case of vulvar schistosomiasis, which falls under female genital schistosomiasis, a neglected tropical disease.

## 2. Case Summary

**Figure 1:**
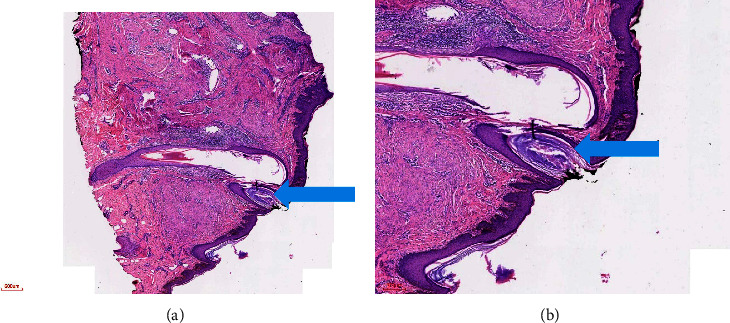
Epidermis and dermis. Haemtoxylin and eosin stain showing pseudohorn cyst (blue arrow) at (a) 2x and (b) 4x magnifications.

**Figure 2:**
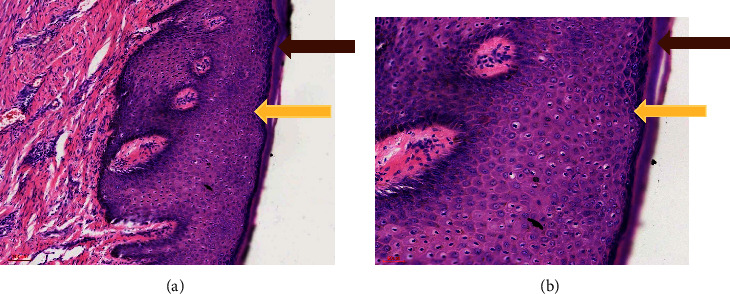
Epidermis. Haematoxylin and eosin stain showing hyperkeratosis (brown arrow) and parakeratosis (yellow arrow) at (a) 10x and (b) 20x magnifications.

**Figure 3:**
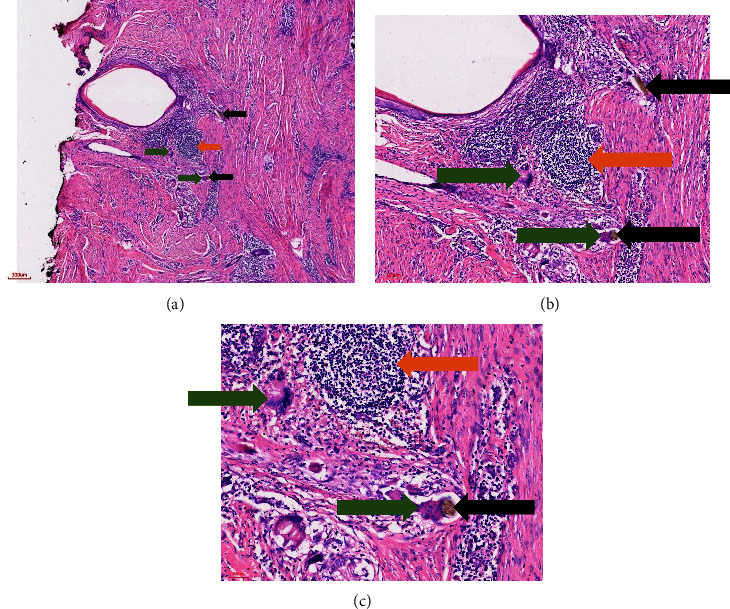
Dermis. Haematoxylin and eosin stain showing schistosome ova (black arrow) engulfed by foreign body giant cells (green arrow) and a collection of mononuclear inflammatory cells (orange arrow) at (a) 4x, (b) 10x, and (c) 20x magnifications.

**Figure 4:**
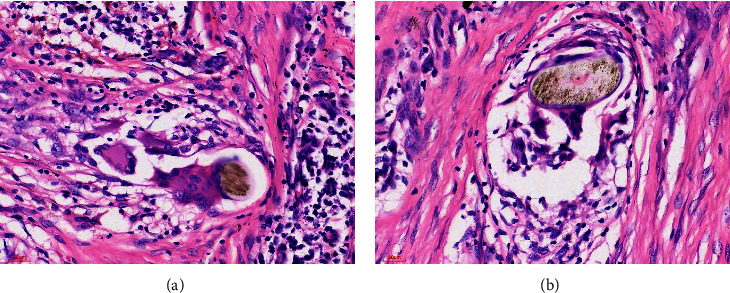
Dermis. Haematoxylin and eosin stain showing a schistosome ovum engulfed by a foreign body of giant cells with a granuloma at 40x magnification.

**Figure 5:**
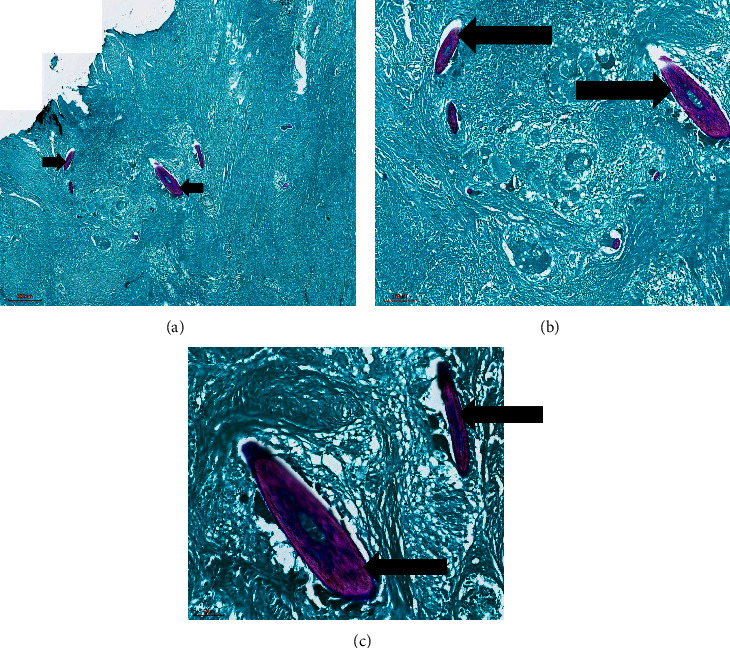
Dermis. Ziel–Neelsen stain showing *S*. *mansoni* ova (black arrow) at (a) 4x, (b) 10x, and (c) 20x magnifications.

A 69-year-old married woman presented to a peripheral primary healthcare facility on multiple occasions with a persistent vulvar itch of 8 months duration, which was not responding to treatment. A clinical diagnosis of vulvar lichen planus unresponsive to medical therapy was made, and a vulvectomy was done for histopathology examination. At the histopathology laboratory, an ellipse of skin measuring 21.0 × 11.0 × 7.0 mm was received. There was ulceration at the epidermal surface, measuring 7.0 mm across the widest diameter. The other surfaces were grossly unremarkable. The cut surfaces of the lesion were whitish in colour and solid in consistency. The tissue was taken through a standard histopathology tissue processing procedure [17]. Microscopic examination after haematoxylin and eosin (H&E) staining revealed skin showing parakeratosis, papillomatosis, and pseudohorn cysts of the epidermis (Figures 1 and 2). The dermis was densely fibro-collagenous with focal areas of sclerosis and collections of lymphocytes and plasma cells. There were multiple areas of multinucleated giant cells with haphazardly arranged nuclei (foreign body giant cells). These giant cells had engulfed shiny golden-brown and well-defined ovoid structures (schistosome, ova) (Figures 3, 4(a), and 4(b)). There was no malignancy seen. Zeil–Neelson (ZN) staining was consistent with *S. mansoni* ova (Figure 5). A diagnosis of chronic schistosomiasis of the vulva was made. The lesion resolved with the administration of oral praziquantel. Upon follow-up and further investigation, the patient was found to be a white-collar worker in the capital city. There was a history in which the patient waded through waterbodies during her travels to different rural, sub-urban, and urban areas of Ghana. She was positive for a human immunodeficiency virus infection. The stool of the patient was not examined for schistosoma ova, as a lesion resolved on medication, and vulvar schistosomiasis diagnosis is not dependent on stool examination. The following photomicrographs show various areas of the lesion.

## 3. Discussion

This is a case of vulvar schistosomiasis caused by *S. mansoni*, diagnosed in an immunocompromised elderly female with a history of wading through waterbodies during work-related travels. In Ghana, a lot of studies have been conducted on the endemicity of schistosomiasis in the Volta River basin, compared to urbanized areas like Accra [18]. *S. haematobium* is widely distributed across the country; while *S. mansoni* is focally distributed mainly in the former Upper East and West Regions and Volta Region [19]. Due to the endemicity of schistosomiasis in Ghana, the diagnosis of vulvar schistosomiasis in a Ghanaian resident is not unusual. However, only one case of vulvar schistosomiasis caused by *S. haematobium* in an immunocompromised person residing in Ghana was recently published [13].

In Ghana, our case is the first report of vulvar schistosomiasis in an immunocompromised individual caused by *S. mansoni*. Clinically, vulvar schistosomiasis presents as chronic pruritus of the vulva. Our case is similar to that reported by Derkyi-Kwarteng et al. where symptoms persisted for 12 months [13]; in this current case, the symptoms persisted for 8 months. In the *S. haematobium* case, the vulvar itch was associated with ulceration and nonoffensive vaginal watery discharge of 3 months duration [13]. In both cases, symptoms resolved after histopathology diagnosis and treatment with oral praziquantel.

*Schistosoma mansoni* is amongst the species of Schistosoma that are endemic in Ghana and sub-Saharan Africa [2, 3, 16]. Globally, about 250 million people are affected by schistosomiasis, with sub-Saharan Africa accounting for 93% to 97% of the disease burden [10, 20–22]. A cross-sectional study conducted amongst women and girls in 15 riparian communities in Ghana showed that 25.3% and 10.6% had urinary and female genital schistosomiases, respectively [23]. In a retrospective study conducted in the Korle Bu Teaching Hospital (KBTH), Ghana, from 2004 to 2011, the prevalence of tissue schistosomiasis was 0.4% [14]. Schistosomiasis is a devastating disease of the poor [1, 10, 15].

Signs and symptoms of urinary schistosomiasis are well-known, however, those of genital tract schistosomiasis are relatively less known and they are mostly misdiagnosed [24, 25]; even though in Schistosoma edemic areas genital schistosomiasis is not uncommon [10, 23]. Males and females can develop genital tract schistosomiasis [14, 24]. In Ghana, out of 151 tissue schistosomiasis surgical specimens reviewed at the Department of Pathology, KBTH, from 2004 to 2011, 17 (11.3%) were cases of genital schistosomiasis; and 10 (66.7%) out of the 17 were cases of female genital schistosomiasis [14]. Amongst these 17 cases, none was a case of vulvar schistosomiasis and 50% were cases of fallopian tube schistosomiasis [14]. The cervix is the commonest site for female genital schistosomiasis (FGS), followed by the vagina, vulva, ovary, and less commonly, fallopian tube and uterine corpus [26]. FGS presents as vaginal discharge which may be bloody, postcoital bleeding, genital itch and burning sensation, and dyspareunia [27]. It may be complicated by miscarriage, subfertility, ectopic gestation, genital ulcers, and swellings and tumours of the vulva, vagina, and cervix [16, 27, 28].

In 2012, there was a reported case of subfertility and ectopic gestation due to genital schistosomiasis [28]. FGS may be associated with nonspecific symptomatology and mostly misdiagnosed as a sexually transmitted infection [29]. Furthermore, the community and some healthcare professionals may not suspect FGS, although it causes physical and socially debilitating complications [23, 25]. Studies have shown that FGS increases the risk of contracting human immunodeficiency virus (HIV) and human papilloma virus (HPV) [24, 25].

The gold standard for diagnosing vulvar schistosomiasis (VS) is visualizing the tissue presence of schistosome ova in histopathology [1, 5, 16, 30]. Diagnosing VS is characterized by challenges [31]. In histopathology diagnosis with haematoxylin and eosin stain, identification of *schistosoma* species implicated in tissue schistosomiasis is done by visualizing the position of the spines of the ova [32]. *S. haematobium* ovum has a terminally located spine, and *S. mansoni* has a laterally located spine [2, 32]. However, sometimes tissue processing damages the spines, making it difficult to visualize and ultimately determine the specie of schistosoma [32]. Ziehl–Neelsen (ZN) staining is able to overcome this limitation, as *S. mansoni* ova stains pink/red (Figure 5) and *S. haematobium* ova stains green/blueish green like the surrounding tissue [32]. ZN staining was employed in this current case as the spines were not easily seen. Furthermore, urine and stool microscopy for schistosome ova is not appropriate for diagnosing vulvar schistosomiasis or any other female genital schistosomiasis [23, 24, 33]. Detection of schistosome ova in urine or stool is not diagnostic of vulvar schistosomiasis, and the absence of the ova in urine or stool does not exclude vulvar schistosomiasis. Most genital schistosomiases are diagnosed without any urine schistosome ova [31].

Vulvar schistosomiasis increases the risk of contracting human immunodeficiency virus (HIV), human papillomavirus (HPV), and other sexually transmitted infections (STIs) like chlamydia and Neisseria gonorrhea [1, 15, 16, 24, 25, 33]. It is appropriate to investigate patients with STIs when managing patients diagnosed with VS and other FGS [25] as has been demonstrated by the two cases from Ghana. Genitourinary schistosomiasis may also undergo malignant transformation [1, 14].

The treatment of VS is praziquantel [1, 16]. Some laboratory experiments reveal that praziquantel resistance may be emerging in schistosomiasis-endemic regions [33]. There are other antiparasitic agents like oxamniquine for *S. mansoni*, metrifonate for *S. Haematobium*, and artemether for juvenile schistosomes [33]. World Health Organization (WHO) recommends primary and secondary prevention, in addition to praziquantel as chemotherapy [16]. Primary prevention entails avoiding contact with contaminated water [16]Secondary preventions entail routine and mass distribution of praziquantel to at risk populations [16].

## 4. Conclusion

Vulvar schistosomiasis must be considered as a differential diagnosis in patients from Schistosoma endemic areas like Ghana, especially patients from riparian communities who present with itchiness, skin changes, and/or ulcerations of the vulva. Histopathology is the gold standard for diagnosing vulvar schistosomiasis. Comprehensive history and a high index of suspicion are necessary for diagnosing vulvar schistosomiasis. Ziel–Neelsen staining is an ancillary test that can be used to determine the species of schistosome ova.

## 5. Recommendation

Public health programmes must be expanded to target vulvar schistosomiasis and other female genital schistosomiasis because they are silent threats to individual wellbeing.

## Data Availability

All data supporting this report are included in this report.
